# A Stochastic Damage Model for Bond Stress-Slip Relationship of Rebar-Concrete Interface under Monotonic Loading

**DOI:** 10.3390/ma12193151

**Published:** 2019-09-26

**Authors:** Xiaoyong Lv, Zhiwu Yu, Zhi Shan, Ju Yuan

**Affiliations:** 1 School of Civil Engineering, Central South University, Changsha 410075, China; lvxiaoyong@csu.edu.cn (X.L.); zhwyu@csu.edu.cn (Z.Y.); YJcsu0308@163.com (J.Y.); 2 National Engineering Laboratory for High Speed Railway Construction, Changsha 410075, China; 3 Engineering Technology Research Center for Prefabricated Construction Industrialization of Hunan Province, Changsha 410075, China

**Keywords:** rebar-concrete interface, bond stress-slip relationship, stochastic behavior, damage model, genetic algorithm, variance

## Abstract

The stochastic bond stress-slip behavior is an essential topic for the rebar-concrete interface. However, few theoretical models incorporating stochastic behavior in current literature can be traced. In this paper, a stochastic damage model based on micro-mechanical approach for bond stress-slip relationship of the interface under monotonic loading was proposed. In order to describe the mechanical behaviors of the rebar-concrete interface, a microscopic damage model was proposed. By introducing a micro-element consists of parallel spring element, friction element and a switch element, the model is formulated. In order to reflect the randomness of the bond stress-slip behavior contributed by the micro-fracture in the interface, a series of paralleled micro-elements are adopted with the failure threshold of individual spring element is set as a random variable. The expression of both mean and variance for the bond stress-slip relationship was derived based on statistical damage mechanics. Furthermore, by utilizing a search heuristic global optimization algorithm (i.e., a genetic algorithm), parameters of the proposed model are able to be identified from experimental results, which a lognormal distribution has adopted. The prediction was verified against experimental results, and it reveals that the proposed model is capable of capturing the random nature of the micro-structure and characterizing the stochastic behavior.

## 1. Introduction

Although concrete is the most widely material in construction field around world, the bond stress-slip behavior of the rebar-concrete interface has not been fully understood. Among the several focused topics towards the bond stress-slip behavior, the damage of the interface is an essential issue, which has been found in conclusion to be the main reason for degradation of concrete structures [1,2]. Thus, misestimating could be occurred regarding safety and economic design/analysis of relevant structures when consideration of such behavior is absent.

The complexity of bond stress-slip behavior for rebar-concrete interface commonly originated from two essential characteristics; that is, the nonlinearity and randomness. In recent decades, the nonlinearity for bond stress-slip behavior is treated as a fundamental problem, which the constitutive relationship has drawn intensified study and several celebrated works have been conducted by researchers. However, referring to the randomness, research on stochastic properties of relevant material is comparatively blank. In detail, the causes for such behavior can be mainly attributed to the complex inhomogeneous microstructure of the interface, due to the irregular shape, random size and distribution of aggregates, the sedimentation of cement particles, the accumulation of pore water, the curing conditions-induced undetermined hydration, the random corrosion of the rebar, the random size of the ribs, the different location of the concrete and rebar in the structures, and the varied skill of the workers.

Presently, unlike the concrete materials, research on the mechanical behaviors of bond stress-slip to the rebar-concrete interface is relatively rare. Besides, few literature evolved with the theoretical model regarding stochastic behavior can be traced. From a summary standpoint, currently, the researches refer to the theoretical model of the mechanical behaviors of bond stress-slip are able to be classified into three main categories: empirical models, theoretical analysis models and macro-mechanical models.

In detail, the empirical models are usually developed based on the observation of the experimental results, and could also be sub-divided into two types: the segmental function model and continuous function model. Precisely, the most representative contributions of the segmental function models listed in this work were conducted by Alsiwat, et al. [3], Haraji, et al. [4], Eligehausen, et al. [1], Xu [5] and Wang, et al. [6]. For example, Xu [5] divided the bond stress-slip curves into five sections: micro-slip section, slip section, splitting section, descending section and residual section, and thus, the segmental function model of the corresponding sections was proposed. In addition, the representative works of continuous function models were developed by Lutz and Gergely [7], Nilson [8], Mirza and Houde [9], Kankam [10], Di [11], Jin, et al. [12] and Teng, et al. [13]. Although most of the empirical models are able to describe the bond stress-slip behavior, the comprehensive micro-damage mechanism in a physical sense was absence to some extent.

Although aforementioned empirical models may have the advantage of simplifying relative design/analysis process towards certain problems, such formulated equations can be only used for representing specific observed phenomenon. Therefore, a number of theoretical attempts have also been devoted to the modelling of the bond stress-slip behaviors. For instance, considering the force balance of the interface under loading, the stress-strain relationship of the steel bars and concrete and the relationship between slip and strains, Somayaji and Shah [14] proposed the differential equations, corrected the solution of these equations, and obtained the relation model between the local slip and the buried rebar length. Yankelevsky [15] developed a model for interface between un-cracked concrete and deformed steel bars, derived the second-order differential equations dependent on the tensile force of the steel bar, combined the equations with the boundary conditions, further the relationship between bond stress and distance in the longitudinal direction of steel bars was predicted. Zhao and Xiao [16] developed a bond stress-slip model before peak bond stress, formulated based on a wedge-shaped model and mechanism for the interface. Song and Zhao [17] proposed a model based on the stress balance, deformation coordination, and physical conditions by considering the influence of material characteristics, concrete layer thickness, crack spacing, and distance from the crack section. The theoretical analysis models [14,15,16,17,18] considered the micro mechanisms of the interface behaviors, however, they are too complex to apply to engineering including finite element analysis. Nevertheless, there are a number of certain conditional assumptions for the model development. Unfortunately, these assumptions generally did not consider the random nature of the interface micro-structures.

Besides the above-mentioned two categories of theoretical models, the establishment of macroscopic mechanical models were also attracting extensive attentions from researchers. For instance, Alfano, et al. [19,20] established a thermodynamic-based cohesive zone model considering the damage-friction evolution with unilateral contact, which can be used for analysis of rebar-concrete interfaces and cracks in concrete-rock foundation interfaces of concrete dams, etc. Based on continuum damage mechanics and considering the accumulation of interfacial damage of reinforced concrete, Soh, et al. [21] developed a bond stress-slip constitutive model by adopting Weibull distribution function. In view of above introduced models, macroscopic mechanical models, especially the continuum damage mechanical models have been gradually focused by relevant researchers in recent. These models have considered different mechanisms for interface deterioration of reinforced concrete (e.g., damage, friction and interlocking mechanism) based on thermodynamics. However, even though the nonlinearity was effectively addressing and modelling by such models, they are still in deficiency of describing the stochastic properties exhibited in the bond stress-slip behavior.

Therefore, this work aims at developing a stochastic damage constitutive model for bond stress-slip relationship of the rebar-concrete interface in order to capture the random nature of the interface and characterize the stochastic constitutive behaviors. The outline is listed as follows, the characteristics of the microscopic element model of rebar-concrete interface is first introduced, and then a stochastic damage model is proposed based on such microscopic model. Afterward, the expressions of mean and variance of bond stress-slip relationship is derived in Section 2. In addition, random variable parameter identification is conducted by adopting a search heuristic global optimization algorithm (i.e., a genetic algorithm), which the detailed illustration is listed in Section 3. In Section 4, the proposed model is verified against experimental results. Our conclusions are finally given in Section 5.

## 2. Stochastic Damage Model of Bond Stress-Slip Relationship

### 2.1. Microscopic Element Model

**Figure 1:**
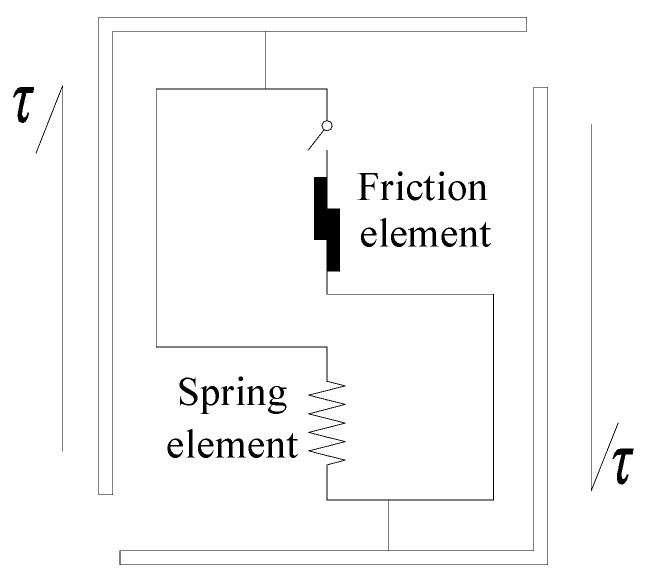
Microscopic element model.

For the purpose of mimicking the mechanical properties of the interface between the rebars and concrete evolved with the interclocking, damage (cracking) and friction, a new bond stress-slip model was proposed based on microscopic elements (see Figure 1). Concretely, the single microscopic element consists of a spring element and a friction element. Therefore, the elastic deformation, debonding and damage (cracking) is able to be modelled by the spring element and the mutual friction and slipping behavior can be characterized by the friction element.

It is assumed that when external load is applied on the interface, the spring element initially undergoes an elastic deformation process, and then breaks (debonding) when the deformation reaches its fracture threshold. As a result, the friction element then experiences a slipping process, which represents the friction between the rebar and concrete. A switch element was also introduced for controlling the work of the spring element and friction element. During the elastic deformation process, the switch element is in opening state, which reveals that the friction element was isolated. After the failure of the spring element, the switch element was closed and caused the friction element began to slide. Therefore, the entire response of the interface are able to be characterized when subject to external loading. Physically, it is worthy of note that such a switch element is not a mechanical element and the aim for introducing it is only for purpose of demonstrating the transformation process from debonding to the frictional sliding in microscopic scale.

Specifically, in this work, in order to describe the randomness of micro-fracture behaviors in the interface between the rebar and concrete, the random variable theory is applied. By presuming the fracture threshold Δ of spring elements obey certain probability densities, the randomness of the failure events are able to be depicted. Therefore, the state of the switch element (open or close) can be determined by comparison between the value of relative displacement between the rebars and concrete *s* and the fracture threshold Δ. That is, when *s* < Δ, the switch element is in an open state, otherwise it is closed.

Hence, the control function of the switch element can be expressed as

(1) $$SWITCH=H(s-\Delta )=\{\begin{array}{c} 1\;\;\;\;s\geq \Delta ,\mathrm{switch}\;\mathrm{on}\;\\ 0\;\;\;\;s<\Delta ,\mathrm{switch}\;\mathrm{off}\; \end{array}$$

where *H* (*x*) is the Heaviside function, *s* is the slip.

### 2.2. Mechanical Behavior of Individual Microscopic Element

Thus, the bond stress-slip relationship for individual microscopic element is able to be divided into two phases:

i. Before the failure of the spring element,

(2) $$\tau_{e}=Gs,s<\Delta$$

where *G* is the stiffness of the bond-slip relationship.

ii. After the failure of the spring element,

(3) $$\tau_{r}=\beta \tau_{u}=\beta G\Delta ,s\geq \Delta$$

where *β* is the coefficient of friction and *τ*_u_ is the failure stress of the spring *τ*_u_ = *G*Δ.

**Figure 2:**
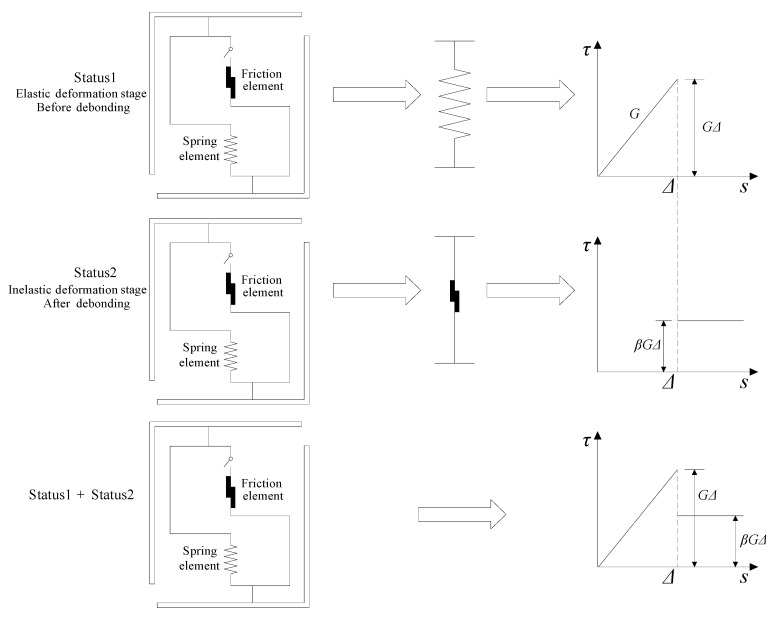
Sketch of two statuses of individual microscopic element under loading.

Therefore, the entire bond stress-slip relationship of individual microscopic element can be expressed as follows (see Figure 2):

(4) τ=H(Δ−s)Gs+βGΔH(s−Δ)

### 2.3. Mechanical Behavior of Parallel System of Microscopic Elements

**Figure 3:**
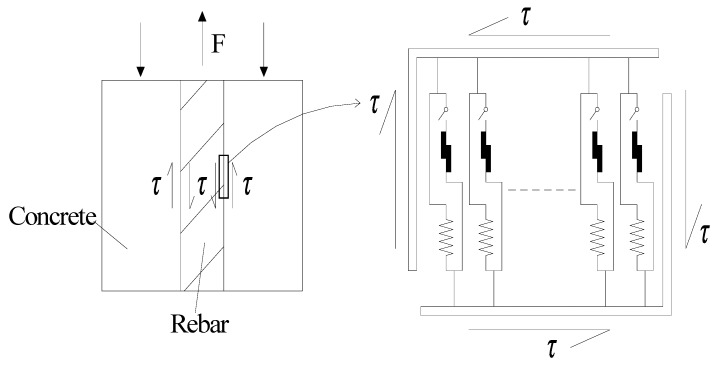
Sketch of parallel elements system.

In order to mimic the corresponded relationship of the rebar-concrete interface, a representative interface element (RIE) in mesoscale was firstly introduced in this work, which consists of a parallel system of microscopic elements (see Figure 3). In detail, based on Equation (4), the bond stress of the *k*-th microscopic element was defined such that (*k* = 1, 2, 3, ···, *N*)

(5) $$\tau_{k}=H[\Delta \left(x_{k}\right)-s]G_{k}s+H[s-\Delta (x_{k})]\beta_{k}G_{k}\Delta$$

where *x_k_* denotes the location of the *k*-th microscopic element in the coordinate.

By assuming that the stiffness *G* and the coefficient of friction *β* of individual element is equal, the average bond stress for parallel system is obtained as follows:

(6) $$\tau =\frac{1}{N}\sum_{k=1}^{N}H[\Delta (x_{k})-s]Gs+\frac{1}{N}\sum_{k=1}^{N}H[s-\Delta (x_{k})]\beta G\Delta (x_{k})$$

Taking the limit of Equation (6) as *N* approaches infinity, one obtains

(7) $$\tau =\int_{0}^{1}H[\Delta (x)-s]Gsdx+\int_{0}^{1}\beta G\Delta H[\Delta (x)-s]dx$$

**Figure 4:**
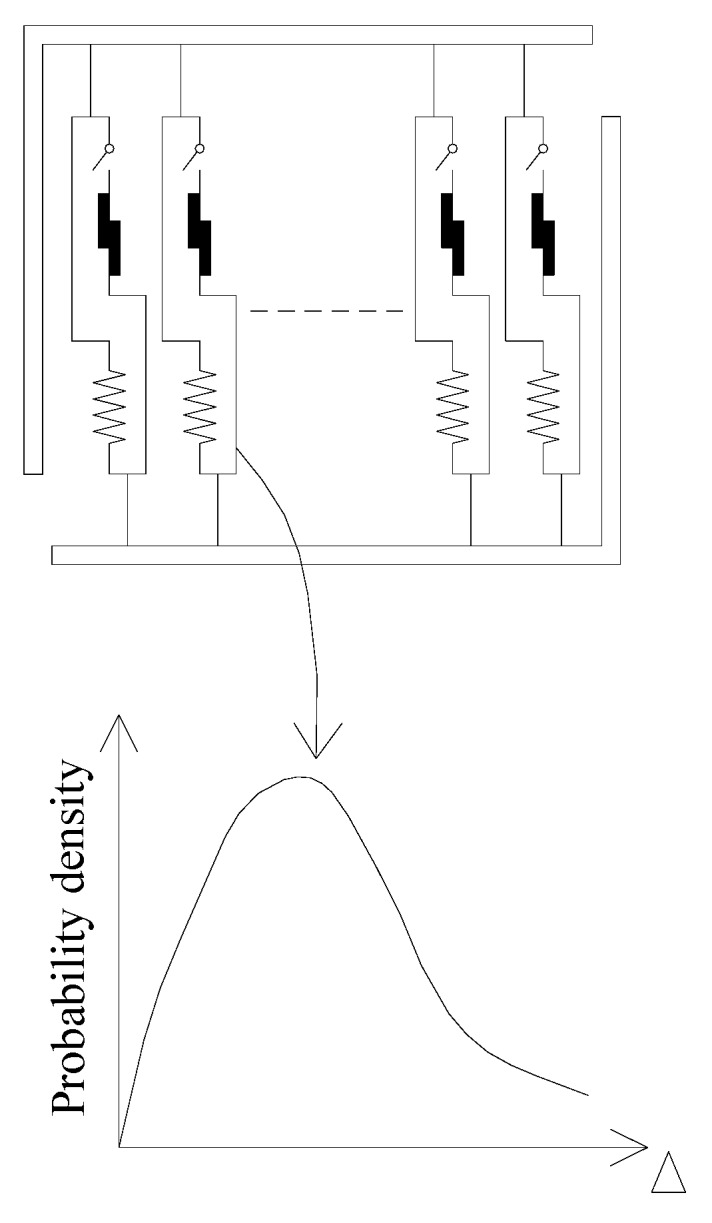
Sketch of the fracture threshold.

In addition, the fracture threshold Δ is set as a random variable, with density function *f*(Δ) and distribution function *F*(Δ) = ʃ_0_^+^^h^*f*(Δ)dΔ, as illustrated in Figure 4.

Since the failures of spring element represent the damage of the interface, the damage variable can be defined as the ratio of damage area to total area of the parallel system [22,23] as follows:

(8) $$d=\frac{A_{d}}{A}=\frac{1}{N}\sum_{k=1}^{N}H\left[s-\Delta (x_{k})\right]$$

When *N* approaches to infinity, the damage variable is able to be obtained as follows,

(9) $$d(s)=\int_{0}^{1}H[s-\Delta (x)]\;dx$$

By substituting Equation (9) to Equation (7), a damage model for the bond stress-slip relationship is derived such that

(10) $$\tau =(1-d)Gs+\beta G\int_{0}^{1}\Delta H(s-\Delta )\;dx$$

especially if the damage variable *d* = 0 implies the interface is in a non-damage state and *τ = Gs*. When the damage variable *d* = 1, it represents that the interface is completely damaged and only residual frictional stress is existed, that is, *τ* =*βG* ∫_0_^1^ Δ(*x*)d*x*.

Hence, the mean value of the damage variable *d* can be expressed as follows:

(11) $$E[d(s)]=\int_{0}^{\infty }\int_{0}^{1}H[s-\Delta (x)]f(\Delta )dxd\Delta =\int_{0}^{1}\int_{0}^{s}f(\Delta )dxd\Delta =\int_{0}^{1}F(s)dx=F_{\Delta }(s)$$

The square of the mean value of the damage variable is derived such that,

(12) $$\begin{array}{l} E[d^{2}(s)]=\int_{0}^{\infty }\int_{0}^{\infty }\left[\int_{0}^{1}\int_{0}^{1}H(s-\Delta_{1})H(s-\Delta_{2})dx_{1}dx_{2}\right]f_{\Delta }(\Delta_{1},\Delta_{2})d\Delta_{1}d\Delta_{2}\\ =\int_{0}^{1}\int_{0}^{1}\left[\int_{0}^{s}\int_{0}^{s}f_{\Delta }(\Delta_{1},\Delta_{2})d\Delta_{1}d\Delta_{2}\right]dx_{1}dx_{2}\\ =\int_{0}^{s}\int_{0}^{s}f_{\Delta }(\Delta_{1},\Delta_{2})d\Delta_{1}d\Delta_{2} \end{array}$$

where *f*_Δ_(Δ_1_, Δ_2_) is the two-dimensional joint probability density function of the fracture threshold for spring elements.

### 2.4. Mean Value of Stochastic Mechanical Responses

Considering the properties between the expected operator and the integral operator, the following results can be obtained:

(13) $$\begin{array}{l} \mu_{\tau }(s)=\mu [\int_{0}^{1}H\left(\Delta -s\right)Gsdx+\int_{0}^{1}\beta G\Delta H\left(s-\Delta \right)dx]\\ \;\;\;\;\;=\mu \left[\int_{0}^{1}H\left(\Delta -s\right)Gsdx\right]+\mu \left[\int_{0}^{1}\beta G\Delta H\left(s-\Delta \right)dx\right]\\ \;\;\;\;\;=\int_{0}^{1}\mu \left[H\left(\Delta -s\right)Gs\right]dx+\int_{0}^{1}\mu \left[\beta G\Delta H\left(s-\Delta \right)\right]\;dx \end{array}$$

where

(14) $$\begin{array}{l} \mu \left\{H\left[s-\Delta \left(x\right)\right]\right\}\\ =1\times P\left[H\left(s-\Delta \left(x\right)\right)=1\right]+0\times P\left[H\left(s-\Delta \left(x\right)\right)=0\right]\\ =P\left[H\left(s-\Delta \left(x\right)\right)=1\right]\\ =P\left[s-\Delta \left(x\right)\geq 0\right]=P\left[\Delta \left(x\right)\leq s\right]\\ =\int_{0}^{s}f\left(\Delta \right)d\Delta =F\left(s\right) \end{array}$$

(15) $$\mu \left\{H\left[\Delta \left(x\right)-s\right]\right\}=1-\int_{0}^{s}f(\Delta )d\Delta =1-F(s)$$

*P* denotes the probability.

Therefore, the mean function of bond stress can be expressed as

(16) $$\begin{array}{l} \mu_{\tau }\left(s\right)=\int_{0}^{1}\mu \left[H\left(\Delta -s\right)Gs\right]dx+\int_{0}^{1}\mu \left[\beta G\Delta H\left(s-\Delta \right)\right]dx\\ =Gs\int_{0}^{1}\left(1-\int_{0}^{s}f\left(\Delta \right)d\Delta \right)dx+\beta G\int_{0}^{1}\int_{0}^{s}\Delta f\left(\Delta \right)d\Delta dx \end{array}$$

### 2.5. Variance Value of Stochastic Mechanical Responses

The variance function of stochastic mechanical responses of the parallel system is expressed as follows,

(17) $$V^{2}\left(\tau \right)=E\left(\tau^{2}\right)-E^{2}\left(\tau \right)$$

By combining Equations (9) and (10), it is able to obtain *E*(*τ*^2^) such that,

(18) $$\begin{array}{l} E\left(\tau^{2}\right)=E\left\{{\left(1-d\right)}^{2}G^{2}s^{2}+\beta^{2}G^{2}{\left[\int_{0}^{1}\Delta H\left(s-\Delta \right)dx\right]}^{2}+2\beta G^{2}s\left(1-d\right)\int_{0}^{1}\Delta H\left(s-\Delta \right)dx\right\}\\ =G^{2}s^{2}\left[1+E\left(d^{2}\right)-2E\left(d\right)\right]+\beta^{2}G^{2}\int_{0}^{\infty }\int_{0}^{\infty }\left[\int_{0}^{1}\Delta_{1}H\left(s-\Delta_{1}\right)dx_{1}\int_{0}^{1}\Delta_{2}H\left(s-\Delta_{2}\right)dx_{2}\right]f_{\Delta }\left(\Delta_{1},\Delta_{2}\right)d\Delta_{1}d\Delta_{2}\\ =2\beta G^{2}s\left\{\int_{0}^{\infty }\int_{0}^{1}\Delta H\left(s-\Delta \right)f\left(\Delta \right)dxd\Delta -\int_{0}^{\infty }\int_{0}^{\infty }\left[\int_{0}^{1}\Delta H_{1}\left(s-\Delta_{1}\right)dx_{1}\int_{0}^{1}H\left(s-\Delta_{2}\right)dx_{2}\right]f_{\Delta }\left(\Delta_{1},\Delta_{2}\right)d\Delta_{1}d\Delta_{2}\right\} \end{array}$$

By substituting Equations (11) and (12) to Equation (18), *E*(*τ*^2^) is able to be derived as follows

(19) $$\begin{array}{l} E(\tau^{2})=G^{2}s^{2}\left[1+\int_{0}^{s}\int_{0}^{s}f_{\Delta }(\Delta_{1},\Delta_{2})d\Delta_{1}d\Delta_{2}-2\int_{0}^{s}f\left(\Delta \right)d\Delta \right]+\beta^{2}G^{2}\int_{0}^{s}\int_{0}^{s}\Delta_{1}\Delta_{2}f_{\Delta }\left(\Delta_{1},\Delta_{2}\right)d\Delta_{1}d\Delta_{2}\\ +2\beta G^{2}s\int_{0}^{s}\Delta f\left(\Delta \right)d\Delta -2\beta G^{2}s\int_{0}^{s}\int_{0}^{s}\Delta_{1}f_{\Delta }\left(\Delta_{1},\Delta_{2}\right)d\Delta_{1}d\Delta_{2} \end{array}$$

Therefore, by substituting Equations (16) and (19) to Equation (17), the variance value of the stochastic mechanical responses is able to be obtained as follows,

(20) $$\begin{array}{l} V^{2}\left(\tau \right)=G^{2}s^{2}\left[1+\int_{0}^{s}\int_{0}^{s}f_{\Delta }\left(\Delta_{1},\Delta_{2}\right)d\Delta_{1}d\Delta_{2}-2\int_{0}^{s}f\left(\Delta \right)d\Delta \right]+\beta^{2}G^{2}\int_{0}^{s}\int_{0}^{s}\Delta_{1}\Delta_{2}f_{\Delta }\left(\Delta_{1},\Delta_{2}\right)d\Delta_{1}d\Delta_{2}\\ +2\beta G^{2}s\int_{0}^{s}\Delta f\left(\Delta \right)d\Delta -2\beta G^{2}s\int_{0}^{s}\int_{0}^{s}\Delta_{1}f_{\Delta }\left(\Delta_{1},\Delta_{2}\right)d\Delta_{1}d\Delta_{2}-{\left[Gs\int_{0}^{1}\left(1-\int_{0}^{s}f\left(\Delta \right)d\Delta \right)dx+\beta G\int_{0}^{1}\int_{0}^{s}\Delta f\left(\Delta \right)d\Delta dx\right]}^{2} \end{array}$$

By presuming the fracture threshold Δ of obeys the lognormal distribution [22,24] and defining the mean and the variance of the fracture threshold as *μ*_Δ_ and *σ*_Δ_, let *Z*(*x*) be a homogeneous normal function with the mean value and standard deviation (*λ*, *ζ*^2^), it is derived that

(21) Z(x)=lnΔ(x)

(22) $$\lambda =E\left[\ln\Delta \left(x\right)\right]=\ln\left(\frac{\mu }{\sqrt{1+\sigma_{\Delta }^{2}/\mu_{\Delta }^{2}}}\right)$$

(23) $$\zeta^{2}=\mathrm{var}\left[\ln\Delta \left(x\right)\right]=\ln\left(1+\sigma_{\Delta }^{2}/\mu_{\Delta }^{2}\right)$$

Therefore, the distributions can be obtained respectively as follows,

(24) $$f\left(\Delta \right)=\frac{1}{\sqrt{2\pi }\Delta \zeta }\exp\left[-1/2{\left(\frac{\ln\Delta -\lambda }{\zeta }\right)}^{2}\right]$$

(25) $$f_{\Delta }\left(\Delta_{1},\Delta_{2}\right)=\frac{1}{2\pi \zeta_{1}\zeta_{2}\sqrt{1-\rho^{2}}\Delta_{1}\Delta_{2}}\exp\left\{\frac{-1}{2\left(1-\rho^{2}\right)}\left[\frac{{\left(\ln\Delta_{1}-\lambda_{1}\right)}^{2}}{\zeta_{1}^{2}}-2\rho \frac{\left(\ln\Delta_{1}-\lambda_{1}\right)\left(\ln\Delta_{2}-\lambda_{2}\right)}{\zeta_{1}\zeta_{2}}+\frac{{\left(\ln\Delta_{2}-\lambda_{2}\right)}^{2}}{\zeta_{2}^{2}}\right]\right\}$$

Consequently, the properties of the fracture threshold can be described by the parameters *λ*, *ζ*, *λ*_1_, *λ*_2_, *ζ*_1_, *ζ*_2_ and *ρ*.

## 3. Random Variable Parameter Identification Based on Genetic Algorithm

To verify the foregoing analytical model proposed in this work, a random variable parameter identification process is developed based on genetic algorithm. Genetic algorithms (GAs) is a search method relative to the theory of evolution, including the processes of reproduction, crossover, mutation, and selection, create populations of solutions for optimizing an objective function. The applicability of GAs was widely accepted by relevant researchers for dealing with the problems of optimizing analysis.

In detail, by observing the *τ- s* relationship from a group of pullout tests, the mean and variance of *τ- s* curves are able to be determined. Secondly, by discretizing the mean and variance of *τ- s* curves into *i*th intervals, corresponded points (*s_i_,μ(τ_i_^o^))* and (*s_i_,v(τ_i_^o^))* are selected. At last, by calculating the corresponded points (*s_i_,μ(τ_i_))* and (*s_i_,v(τ_i_))* from the proposed model, an objective function *R* is able to be constructed, which is illustrated as follows:

(26) $$R_{\mu }=\sum_{i=1}^{N}{[\mu (\tau_{i})-\mu (\tau_{i}^{o})]}^{2}\overset{approach}{\to }min$$

(27) $$R_{V^{2}}=\sum_{i=1}^{N}{[V^{2}(\tau_{i})-V^{2}(\tau_{i}^{o})]}^{2}\overset{approach}{\to }min$$

**Figure 5:**
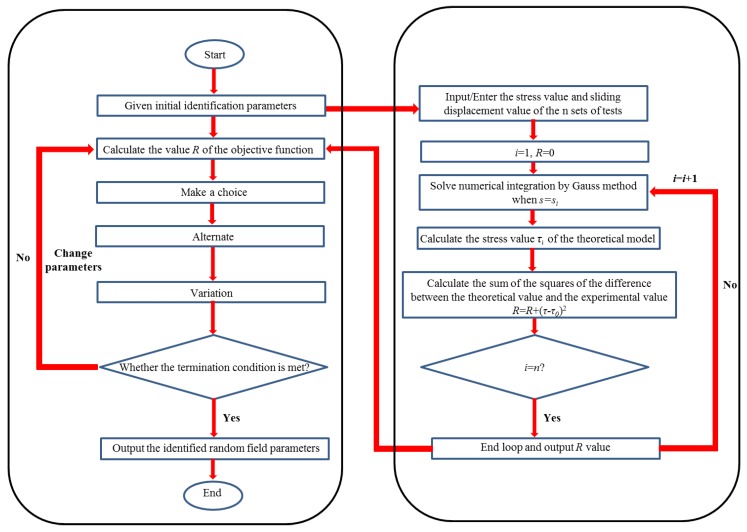
Flow chart of algorithm for parameter identification of random variable. (**a**) Main program module of genetic algorithm; (**b**) Main program module of the objective function

Specifically, an optimization criterion is introduced for determining the applicability of the objective function *R*, that is, the value of *R* is approaching minimum. The identification flow chart is listed in Figure 5.

## 4. Model Validation and Discussion

### 4.1. Bond Stress-Slip Behaviors in the Sense of Mean Value

**Figure 6:**
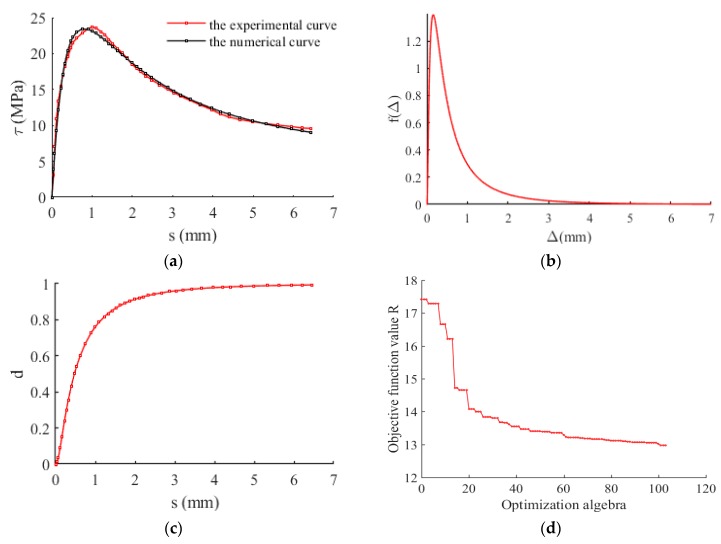
Bond stress-slip behavior in the sense of mean value. Experimental results were obtained from literature [25]. (**a**) Comparison between experimental and predicted results; (**b**) The probability density function; (**c**) Damage evolution; (**d**) The graph of objective function *R* changing with optimization algebra.

**Figure 7:**
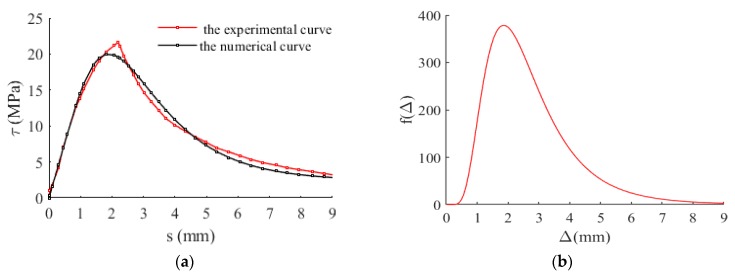

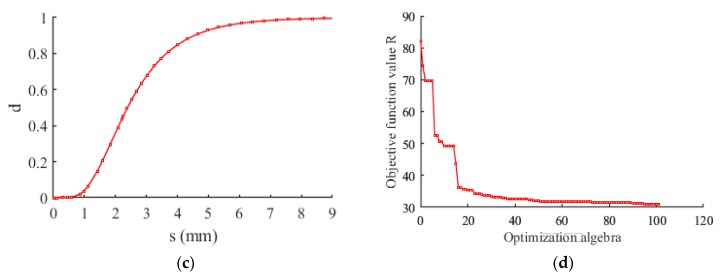
Bond stress-slip behavior in the sense of mean value. Experimental results were obtained from literature [26]. (**a**) Comparison between experimental and predicted results; (**b**) The probability density function; (**c**) Damage evolution; (**d**) The graph of objective function *R* changing with optimization algebra.

To validate the effectiveness of the proposed model for the mean *τ- s* curve in this work, the experimental results of bond stress-slip relationship under monotonic loading [25,26] were used in this section. The parameters of the proposed model were determined by using the above-mentioned random identification process (i.e., a genetic algorithm), and the results were shown in Table 1 and Figure 6, Table 2 and Figure 7, respectively. It is observed from Figure 6 and Figure 7 that the predicted results agrees well with the experimental results.

### 4.2. Stochastic Bond Stress-Slip Behaviors

**Figure 8:**
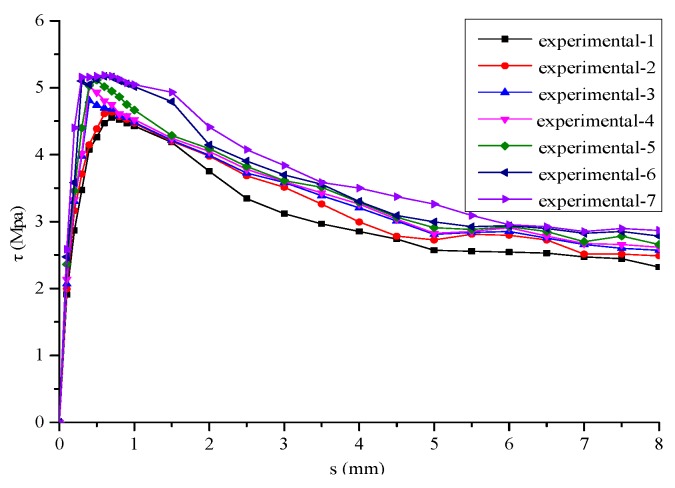
Experimental results of pullout tests [23,24,25].

In order to verify the effectiveness of the proposed model for characterizing the stochastic behaviors, the experimental results of a series of pull-out tests [23,27,28] were examined in this section (see Figure 8).

**Figure 9:**
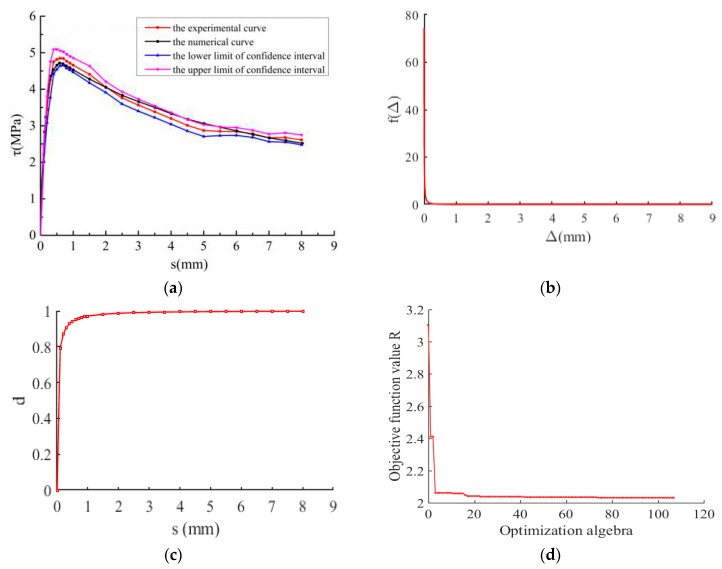
Bond stress-slip behavior in the sense of mean value. Experimental results were obtained from literature [23,27,28]. (**a**) Comparison between experimental and predicted results; (**b**) The probability density function; (**c**) Damage evolution; (**d**) The graph of objective function R changing with optimization algebra.

**Figure 10:**
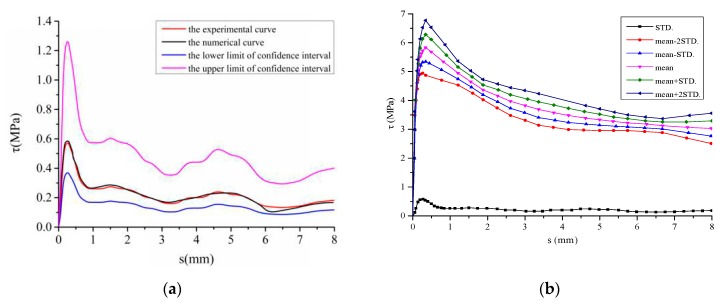

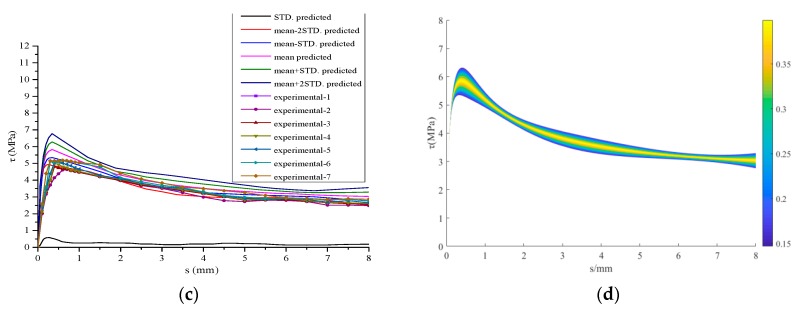
Stochastic bond stress-slip behaviors. (**a**) Comparison between predicted and experimental results for standard deviation value; (**b**) Curves of predicted stochastic behaviors; (**c**) Comparison between predicted and experimental results; (**d**) Cloud map of predicted stochastic behaviors.

By using the proposed identification process, the parameters and predicted results were shown in Table 3 and Table 4 and Figure 9 and Figure 10.

Specifically, Figure 10 illustrates the effectiveness of the proposed model for characterizing the stochastic mechanical behaviors of the interface. It shows that the proposed model enable us to capture the random nature of the interface by the derived expression of the variance, based on the micro mechanics and random variable theory [22,24]. In detail, the proposed model is able to describe both the mean and variance value of the bond stress-slip behaviors (see Figure 6 and Figure 7, Figure 9 and Figure 10) and promotes the development and application of statistical mechanics and random variable theory by deriving the expression of variance in Section 2, Section 3, Section 4 and Section 5. Moreover, the literature [24] has considered that this expression is very difficult to be obtained and further study is needed in the future, due to the complexity of related theoretical analysis.

### 4.3. The Comparison between the Proposed Model with a Non-Stochastic Model

**Figure 11:**
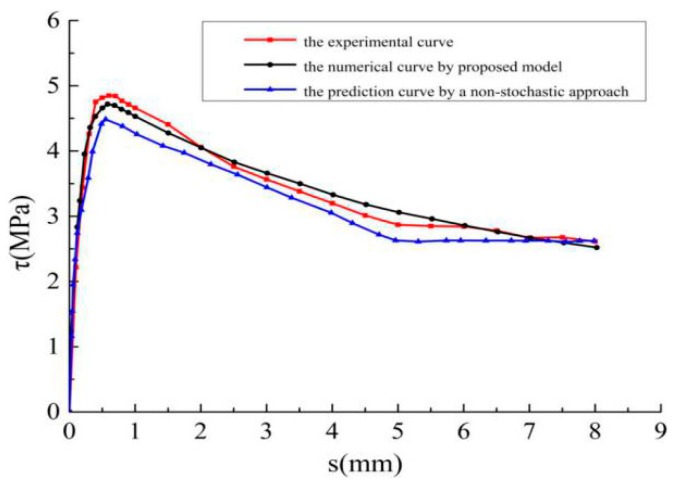
The comparison between the proposed model and a non-stochastic model.

A comparison between the predicted mean *τ–ѕ* curve by using the proposed model and a non-stochastic model obtained by literature [27] is listed in Figure 11. The observation shows that the predicted *τ–s* curve generated by proposed model was closer to the experimental results, while an underestimation of the stress in most load region is found from the prediction obtained by the non-stochastic model. Additionally, by considering a lack of the capability of characterizing the stochastic behaviors (variance), the applicability of the proposed model is more widely to some extent compared with the non-stochastic model mentioned in this work.

## 5. Conclusions

In this paper, a stochastic damage model for bond stress-slip relationship of rebar-concrete interface was proposed based on the micro mechanical method and random variable theory. The conclusions can be drawn as follows:

A microscopic damage model was proposed to mimic the micro-damage mechanical behaviors of rebar-concrete interface under monotonic loading. The model consisted of a spring element and a friction element in parallel, and the failure/friction behavior of the elements was proposed to be controlled by a switching function. When the slip of the spring element reached a certain threshold, failures occurred and resulted in the friction element slipping instantly. In spite of the conventional theoretical model, by setting the fracture threshold of the spring element as a random variable, the stochastic properties were able to be depicted. More precisely, based on the introduced methodology in this work, the derivation of the expressions for the mean and variance of the bond stress-slip relationship were obtained through the help of statistical mechanics.

A search heuristic global optimization algorithm (i.e., genetic algorithm) was adopted to identify the random variable parameters evolved in the proposed model based on the experimental results, and the lognormal distribution was utilized.

The validation of the proposed model was performed by comparison between the predictions and the experimental results. It has been revealed that the proposed model can effectively describe the stochastic constitutive relationship of the bond stress-slip of rebar-concrete interface under monotonic loading.

This work may be of significance for studying and modelling the stochastic constitutive relationship of bond stress-slip relationship of rebar-concrete interface under uniaxial monotonic loading, as well as providing a better understanding and awareness of its uncertain effects on relevant engineering applications.

## Figures and Tables

**Table 1:** Identification results of random variable parameters. Experimental results were obtained from literature [26].

*λ*	*ζ*	*β*	*G*(MPa)	*R*
6.11	1.08	0.07	0.09	12.86

**Table 2:** Identification results of random variable parameters. Experimental results were obtained from literature [26].

*λ*	*ζ*	*β*	*G*(MPa)	*R*
0.872	0.5	0.056	15.25	28.82

**Table 3:** Identification results of random variable parameters of the mean value.

*λ*	*ζ*	*β*	*G*(MPa)	*R*
5.076	1.328	0.102	0.099	2.03

**Table 4:** Identification results of random variable parameters of the variance value.

*λ*_1._	*λ*_2_	*ζ*_1_	*ζ*_2_	*ρ*	*R*
5.049	4.96	1.292	1.358	0.022	3.3
